# Epstein–Barr Virus Promotes Gastric Cancer Progression by Modulating m6A-Dependent YTHDF1–TSC22D1 Axis

**DOI:** 10.3390/microorganisms13122820

**Published:** 2025-12-11

**Authors:** Yea Rim An, Jaehun Jung, Kyeong Min Kwon, Jun Yeob Kim, Min-Hyeok Lee, Ju Yeon Lee, Minho Lee, Suk Kyeong Lee

**Affiliations:** 1 Department of Medical Life Sciences, College of Medicine, The Catholic University of Korea, Seoul 06591, Republic of Korea; 2 Department of Medical Sciences, College of Medicine, The Catholic University of Korea, Seoul 06591, Republic of Korea; 3 Department of Life Science, Dongguk University-Seoul, 32 Dongguk-ro, Ilsandong-gu, Goyang-si 10326, Gyeonggi-do, Republic of Korea

**Keywords:** Epstein–Barr virus, gastric cancer, m6A modification, YTHDF1, TSC22D1, epitranscriptomics

## Abstract

Epstein–Barr virus (EBV) infection is closely associated with gastric cancer, yet its role in m6A-dependent gene regulation remains poorly understood. In this study, we investigated how EBV infection alters the m6A methylation pattern in gastric cancer cells and examined its impact on TSC22D1 mRNA stability through interaction with the m6A reader protein YTHDF1. m6A RNA immunoprecipitation sequencing (MeRIP-seq) revealed a significant reduction in m6A methylation of TSC22D1 in EBV-infected gastric cancer cells (AGS-EBV) compared with EBV-negative cells (AGS). Moreover, YTHDF1 knockdown increased both the stability and expression of TSC22D1. These findings demonstrate that YTHDF1 binds to TSC22D1 mRNA and promotes its m6A-dependent degradation. Collectively, our results suggest that EBV infection modulates m6A modification to regulate gene stability and identify the YTHDF1–TSC22D1 axis as a potential therapeutic target in EBV-associated gastric cancer.

## 1. Introduction

Gastric cancer remains one of the leading causes of cancer-related mortality worldwide and is particularly prevalent in East Asia. According to The Cancer Genome Atlas (TCGA), gastric cancer can be molecularly classified into four subtypes: Epstein–Barr virus–associated gastric cancer (EBVaGC), microsatellite instability (MSI), chromosomal instability (CIN), and genomically stable (GS). Among these, EBVaGC accounts for approximately 9–10% of cases [1,2]. This EBV-positive subtype exhibits distinctive molecular characteristics, including CpG island hypermethylation, modulation of the tumor micro-environment, and altered inflammatory signaling [3,4]. Although EBV infection is well recognized as a contributor to gastric tumorigenesis via DNA methylation and histone modifications [5,6], the full spectrum of mechanisms by which EBV drives malignant progression remains insufficiently understood.

Beyond canonical DNA-level epigenetic regulation, epitranscriptomic modifications have emerged as critical regulators of gene expression in cancer. In particular, N6-methyladenosine (m6A) is the most abundant internal modification in mammalian mRNA, modulating transcript stability, splicing, translation, and degradation [7,8,9]. This modification is dynamically installed by “writers” (e.g., METTL3, METTL14, WTAP), removed by “erasers” (e.g., FTO, ALKBH5) [10,11], and interpreted by “readers” (e.g., YTHDF1, YTHDF2, YTHDF3, IGF2BP family, HNRNPC) [12,13,14]. Among these readers, YTHDF1 has been widely implicated in cancer. By binding m6A-modified transcripts, YTHDF1 can promote translation initiation through interactions with ribosomes or eIF3, thereby increasing expression of oncogenic proteins [15,16]. Elevated YTHDF1 expression has been documented in multiple cancers and is tied to enhanced cell proliferation, migration, and tumor aggressiveness [17,18].

Intriguingly, accumulating evidence suggests that viral infections can themselves exploit m6A pathways. YTHDF family proteins have been shown to bind viral RNAs (e.g., Zika virus, HIV-1), affecting viral RNA stability, translation, and host–virus interactions [19,20]. In the context of EBV, YTHDF1 has been reported to recognize m6A-modified viral transcripts, promoting their decapping and degradation to suppress EBV lytic replication [21]. However, these studies have largely focused on viral RNA–reader interactions. As a result, the potential role of YTHDF1 in modulating host gene expression in EBV-associated gastric cancer remains largely unexplored.

In this study, we investigated how EBV infection remodels m6A-mediated post-transcriptional regulation in gastric cancer, centering on the YTHDF1–TSC22D1 axis. By integrating transcriptome profiling, m6A-RIP sequencing, and functional assays, we show that EBV suppresses YTHDF1 expression in part via BART miRNAs. This suppression reshapes the host m6A landscape, and specifically reduces m6A on TSC22D1 mRNA. The resulting loss of methylation diminishes YTHDF1 binding and increases TSC22D1 mRNA stability, thereby enhancing its oncogenic impact. These findings advance our understanding of EBV-driven epitranscriptomic reprogramming in gastric cancer and reveal novel targets for therapeutic intervention.

## 2. Materials and Methods

### 2.1. Cell Lines and Culture

AGS, SNU-216, and SNU-719 cells were purchased from the Korean Cell Line Bank (Seoul, Republic of Korea). The YCCEL1 cell line was provided by Professor Sun Young Rha (Yonsei University College of Medicine, Seoul, Republic of Korea). SNU-719 and YCCEL1 are naturally EBV-infected gastric cancer cell lines. AGS-EBV cells were provided by Takada K (Institute for Genetic Medicine, Hokkaido University, Hokkaido, Japan). AGS-EBV is a gastric cancer cell line infected with the recombinant Akata EBV strain. All cells were cultured in RPMI 1640 medium supplemented with 10% fetal bovine serum, 1% penicillin-streptomycin, and 0.1% amphotericin B. AGS-EBV cells were maintained in medium containing 400 µg/mL G418. All cells were incubated at 37 °C in a humidified atmosphere with 5% CO_2_.

### 2.2. Clinical Tissue Samples

Fresh-frozen gastric cancer tissue samples were obtained in an anonymized form from the Human Tissue Bank of Seoul St. Mary’s Hospital. Samples collected between the years 2020 and 2025 were confirmed as either EBV-negative or EBV-positive based on pathological examination and immunohistochemical (IHC) staining. The use of human tissue samples was reviewed and determined to be exempt from IRB approval by the Institutional Review Board (IRB) of The Catholic University of Korea, College of Medicine (IRB No. MC25SSA10014). A total of 24 fresh-frozen gastric cancer tissues, including 12 EBV-negative and 12 EBV-positive samples, were used in this study. Each tissue sample was placed in a tube containing beads and 1 mL of RNAiso Plus (#9109, Takara Bio, Shiga, Japan), and then homogenized using a FastPrep-24™ 5 G Homogenizer (MP Biomedicals, Irvine, CA, USA) for 40 s. To prevent RNA degradation caused by heat, the samples were cooled on ice for 1 min before and after sample processing. The homogenization and cooling cycle was repeated twice to ensure complete tissue disruption. Total RNA was then extracted according to the manufacturer’s protocol, and mRNA expression levels were analyzed by qRT-PCR.

### 2.3. RNA Extraction and qRT-PCR

Cells were harvested, and total RNA was extracted using RNAiso Plus (#9109, Takara Bio) according to the manufacturer’s instructions. cDNA was synthesized from 2 µg of total RNA using oligo (dT) primers and M-MLV reverse transcriptase. Real-time PCR was performed using the CFX Opus 96 Real-Time PCR System (Bio-Rad, Hercules, CA, USA) with TOPreal qPCR 2× PreMIX SYBR-Green with low ROX (#RT500M, Enzynomics, Daejeon, Republic of Korea) (see Table 1 for primer sequences). The PCR conditions were 95 °C for 10 min, followed by 35 cycles of 95 °C for 10 s, 60 °C for 30 s, and 72 °C for 30 s. Relative gene expression was calculated using the quantification cycle (Cq) values and GAPDH as an internal loading control.

### 2.4. Western Blot

Cell lysates were subjected to 10% sodium dodecyl sulfate polyacrylamide gel electrophoresis, and separated proteins were transferred onto a polyvinylidene fluoride membrane. The membranes were blocked and probed with the following primary antibodies against YTHDF1 (#86463, Cell Signaling Technology, Danvers, MA, USA; diluted 1:1000), TSC22D1 (#NBP2-98963, Novus Biologicals, Centennial, CO, USA; diluted 1:1000), and β-actin (#4970S, Cell Signaling Technology; diluted 1:1000). β-Actin was used as a loading control. Bound antibodies were detected using HRP-conjugated anti-rabbit secondary antibodies (Cell Signaling Technology, diluted 1:2000). Protein bands were visualized using an enhanced chemiluminescence detection system (Amersham Bioscience, Piscataway, NJ, USA), and the membrane was exposed to X-ray film (Agfa, Mortsel, Belgium).

### 2.5. Transcriptomic Analysis

Total RNA was extracted from EBV-negative and EBV-positive gastric cancer cell lines, followed by mRNA purification using the Dynabeads mRNA Purification Kit (#61006, Invitrogen, Carlsbad, CA, USA). The extracted mRNA was fragmented into 100–200 nt fragments using the NEBNext Magnesium RNA Fragmentation Module (#E6150S, New England Biolabs, Ipswich, MA, USA) and subsequently purified using the Monarch RNA Cleanup Kit (#T2030, New England Biolabs). Library preparation was performed using the TruSeq Stranded Total RNA Library Prep Human/Mouse/Rat Kit (Illumina, San Diego, CA, USA). Next-generation sequencing (NGS) was conducted using the HiSeq X platform at Macrogen (Seoul, Republic of Korea). The sequencing raw data fastq files were analyzed for read quality using FastQC (v0.12.1) and MultiQC (v1.14), and adapter trimming was determined using Flexba (v3.4.0). Read alignment to the reference genome was performed using STAR (v2.7.7a) [22], generating read counts based on mapped transcript locations. The gene annotation file *Homo_sapiens.GRCh38.104.gtf* from Ensembl was used for mapping, and reads mapped to multiple regions were excluded. Differentially expressed gene (DEG) analysis was conducted using edgeR (v.3.42.4) [23] in R Studio (v2023.06.1+524), based on the read counts obtained from the mapping process.

### 2.6. Microarray Analysis

In this study, previously reported microarray data were utilized for analysis. The microarray analysis of AGS and AGS-EBV cell lines was performed using the Sentrix Human-6 v2 Expression BeadChip (Illumina), and the corresponding data have been deposited in the GEO database under the accession number GSE135644 [24]. Additionally, the raw mRNA microarray data from EBV-positive and EBV-negative gastric cancer patient tissues were obtained from a publicly available study (GEO: GSE51575) and reanalyzed to assess the differential expression of YTH family genes according to EBV infection status.

### 2.7. Transfection of siRNA and Overexpression Vector

All siRNAs were purchased from GenePharma (Shanghai, China), and transfection was performed using Lipofectamine RNAiMAX (#13778150, Invitrogen) according to the manufacturer’s protocol. siRNAs were administered at a final concentration of 10 nM (see Table 2 for sequences), and cells were harvested for RNA and protein extraction 24 h post-transfection. For overexpression experiments, two plasmid constructs were used. The YTHDF1 overexpression plasmid was generated by Bionics (Seoul, Republic of Korea) using a pcDNA3.1(+) backbone containing the human YTHDF1 coding sequence (NM_017798.4) and an ampicillin resistance marker. The TSC22D1 overexpression plasmid was custom-designed and synthesized by VectorBuilder (Chicago, IL, USA) using a pRP[Exp] backbone containing the full-length human TSC22D1 open reading frame (NM_183422.4) along with hygromycin and ampicillin resistance markers. Transfections were performed using 2.5 μg of plasmid DNA and Lipofectamine 2000 (#11668019, Invitrogen) following the manufacturer’s instructions. Cells were collected 24 h after transfection for RNA and protein extraction and phenotypic assays. Successful overexpression was confirmed by qPCR and Western blot, and only validated plasmids were used in subsequent experiments.

### 2.8. MTT Assay (Cell Viability Assay)

Cell proliferation was analyzed using an MTT assay (#21795, Cayman Chemical, Ann Arbor, MI, USA). AGS (5 × 10^3^), SNU-216 (1 × 10^4^), AGS-EBV (7.5 × 10^3^), and YCCEL1 (1 × 10^4^) cells were seeded in each well in 96-well plates. Every 24 h after transfection with siRNA or overexpression vectors, 20 μL of MTT solution (5 mg/mL) was added to each well, followed by incubation for 4 h. The medium was then removed, and 100 μL of DMSO was added to each well. After 5 min, absorbance was measured at 595 nm using a Microplate Reader (Bio-Tek, Winooski, VT, USA).

### 2.9. Soft Agar Colony Formation Assay

AGS, SNU-216, AGS-EBV, and YCCEL1 cells were transfected with siRNA or overexpression vectors. After 24 h, a base layer was prepared in a 6-well plate by combining 1% Bacto Agar with 2× RPMI 1640 medium in a 1:1 ratio. Once solidified, 3000 harvested cells per well were suspended in 0.6% agar and overlaid onto the base layer. The cells were incubated at 37 °C for 3 to 6 weeks. Colonies were imaged using a digital camera and quantified with ImageJ (v1.54g) software. All assays were performed in triplicate, and consistent results were obtained across three independent experiments.

### 2.10. Wound Healing Assay

AGS (1 × 10^6^), SNU-216 (2 × 10^6^), AGS-EBV (1.2 × 10^6^), and YCCEL1 (2 × 10^6^) cells were seeded in each well in 6-well plates. When the cells reached 90–95% confluence, a sterile 200 µL pipette tip was used to scratch the cell monolayer, and phosphate-buffered saline (PBS) was used to wash away detached cells and debris. The cells were then transfected with siRNA or overexpression vectors and incubated in RPMI 1640 medium containing 0.1% FBS at 37 °C. The scratched wound area was observed under a microscope immediately after transfection (0 h) and at 24 h and 48 h post-transfection. Images were captured using TCapture (v4.3.0) software, and wound areas were measured with ImageJ (v1.54g) software. All assays were performed in triplicate, and consistent results were obtained across three independent experiments.

### 2.11. Apoptosis Assay

Twenty-four hours post-transfection, cells were treated with trypsin to detach them, followed by two washes with PBS. Apoptosis was assessed using the PE Annexin V Apoptosis Detection Kit I (#559763, BD Biosciences, Franklin Lakes, NJ, USA). Cells were stained according to the manufacturer’s instructions. Analysis was performed using flow cytometry (BD Biosciences). All assays were performed in triplicate, and consistent results were obtained across three independent experiments.

### 2.12. m6A RNA Methylation Quantification (ELISA-Based Method)

m6A RNA methylation was quantified using an ELISA-based method with the EpiQuik m6A RNA Methylation Quantification Kit (#P-9005, Epigentek, Farmingdale, NY, USA). Total RNA was isolated, and 200 ng RNA was bound to the strip wells using the binding solution. After incubation at 37 °C for 90 min, m6A was captured using an m6A-specific antibody, followed by detection with a secondary antibody conjugated to an enzyme. The enzymatic reaction was initiated by adding the colorimetric substrate, and the absorbance was measured at 450 nm using a Microplate Reader (Bio-Tek, Winooski, VT, USA). The color intensity was proportional to the level of m6A RNA methylation, and absolute quantification was achieved using a standard curve provided with the assay kit.

### 2.13. m6A RNA Immunoprecipitation (MeRIP) and m6A Sequencing

mRNA was extracted from EBV-negative and EBV-positive gastric cancer cell lines. The RNA was then fragmented for subsequent analysis. One-tenth of the fragmented RNA sample was retained as the input sample, and MeRIP was performed with the remained RNA using the EpiMark N6-Methyladenosine Enrichment Kit (#E1610S, New England Biolabs). Library preparation and sequencing analysis were conducted for both input and IP samples. Transcriptomic data were analyzed under two conditions: with or without IP, and with or without EBV infection. To eliminate PCR artifacts, samtools (v1.9) [25] was used to remove duplicated reads with identical sequences and lengths. To identify regions with higher transcript expression in IP samples compared to input samples, the MACS2 (v2.2.8) [26] program was used. Peaks with a false discovery rate (FDR) < 0.05 were selected through statistical testing. Peaks from the same condition were combined using the mergePeaks program in HOMER (v4.11). Common motif sequences in the called peaks were analyzed using the findMotifsGenome program in HOMER (v4.11). Differentially methylated regions (DMRs) based on EBV status were calculated using the RADAR R package (v0.2.1) [27] , and only regions meeting the criteria of log2FC = 1 and FDR < 0.05 were selected.

### 2.14. RNA Immunoprecipitation (RIP)

RNA immunoprecipitation (RIP) was conducted using the RIP RNA-Binding Protein Immunoprecipitation Kit (#17-700, Merck, Darmstadt, Germany) according to the manufacturer’s protocol. Cells were washed with PBS and lysed in RIP lysis buffer supplemented with a protease inhibitor cocktail. The lysates were incubated with either YTHDF1 antibody (#17479-1-AP, Proteintech Group) or normal IgG control, together with Protein A/G Magnetic Beads, at 4 °C for 3 h with gentle rotation. RNA-protein complexes were then washed with RIP wash buffer, treated with Proteinase K, and RNA was isolated via phenol-chloroform extraction followed by ethanol precipitation. The immunoprecipitated RNA was subsequently analyzed by qRT-PCR to assess relative transcript enrichment.

### 2.15. UZH1a Treatment

Cells were seeded in 6-well plates to reach 60–70% confluence, followed by treatment with DMSO or UZH1a (#HY-134673A, MedChemExpress, Monmouth Junction, NJ, USA) at concentrations of 10 µM or 20 µM for 24 h. Following treatment, total RNA was extracted, and mRNA expression changes were assessed by qRT-PCR.

### 2.16. RNA Stability Assay

Cells were seeded in 6-well plates to reach approximately 80% confluence after 24 h, followed by treatment with actinomycin D (1 µg/mL, #A9415, Sigma-Aldrich, St. Louis, MO, USA). Cells were harvested at 0, 2, 4, 6, and 8 h post-treatment for total RNA extraction. RNA expression levels were analyzed by qRT-PCR and normalized to GAPDH.

### 2.17. Statistical Analysis

The MTT and RNA stability assay data were analyzed using one-way analysis of variance (ANOVA) followed by Dunnett’s post hoc test, while Student’s *t*-test was used for the other experiments to determine statistical significance. GraphPad Prism (v8.4.2) (GraphPad Software, San Diego, CA, USA) was used to analyze and draw graphs. *p*-values less than 0.05 were considered statistically significant. All results were expressed as means ± standard deviations.

## 3. Results

### 3.1. EBV Infection Suppresses YTHDF1 Expression in Gastric Cancer

**Figure 1:**
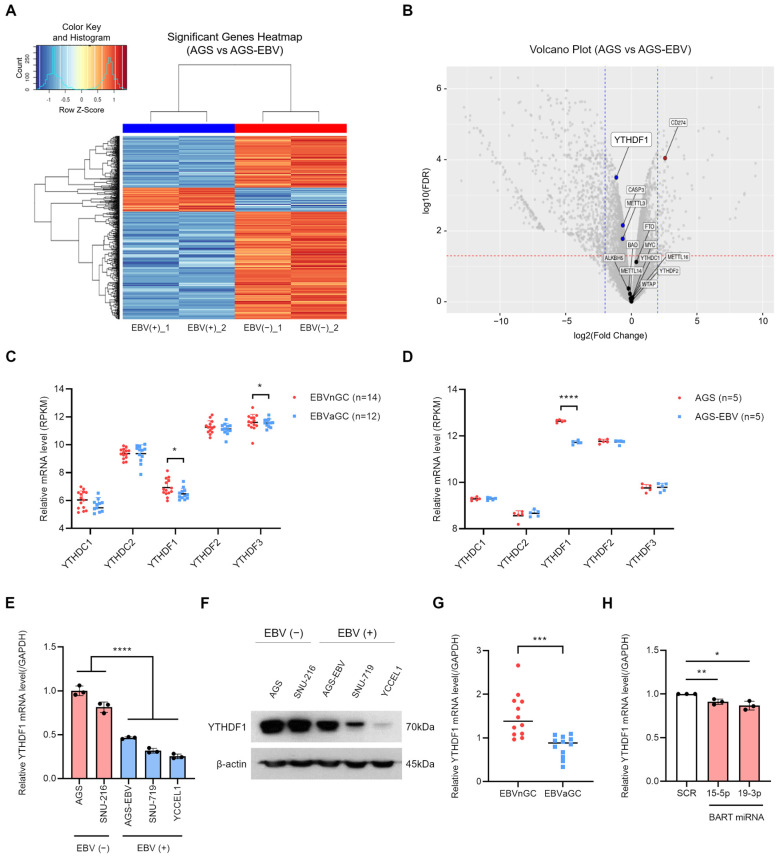
EBV infection reduces YTHDF1 expression in gastric cancer. Differentially expressed genes (DEGs) identified from RNA sequencing analysis of EBV-positive and EBV-negative gastric cancer cells are visualized as a heatmap (**A**) and a volcano plot (**B**). In (B), blue dots indicate significantly downregulated genes, and red dots indicate significantly upregulated genes. The vertical dashed lines indicate the log2(fold change) thresholds (±1), and the horizontal dashed line represents the FDR < 0.05 cutoff. (**C**) The expression levels of YTH family genes in EBV-negative and EBV-positive gastric cancer tissues were analyzed using publicly available microarray data (GEO: GSE51575). (**D**) The expression of YTH family genes was further evaluated using microarray analysis of EBV-negative and EBV-positive gastric cancer cell lines (GEO: GSE135644). YTHDF1 mRNA and protein expression levels were compared in multiple EBV-negative and EBV-positive gastric cancer cell lines using qRT-PCR (**E**) and Western blot analysis (**F**). (**G**) YTHDF1 mRNA expression levels were analyzed by qRT-PCR in EBV-negative and EBV-positive gastric cancer patient tissues. (**H**) qRT-PCR analysis of YTHDF1 mRNA expression in AGS cells transfected with EBV BART miRNA mimics (miR-BART15-5p and miR-BART19-3p) relative to the scramble control. *p*-values: * *p* < 0.05; ** *p* < 0.01; *** *p* < 0.001; **** *p* < 0.0001.

To investigate how EBV infection influences m6A modification, we compared AGS gastric cancer cells and their EBV-infected counterpart, AGS-EBV. These two cell lines are genetically identical except for EBV status. Differentially expressed gene (DEG) analysis revealed 839 significantly altered genes among 15,109 total genes, as shown in the heatmap (Figure 1A) and volcano plot (Figure 1B). Among m6A-related genes, YTHDF1 expression was notably reduced in EBV-positive cells compared with EBV-negative cells (Figure 1A,B). To extend these findings, we analyzed microarray data from 14 EBV-negative and 12 EBV-positive gastric cancer tissues (GEO: GSE51575). Expression of YTHDF1 and YTHDF3 was significantly decreased in EBV-positive tumors relative to EBV-negative tumors (Figure 1C). In contrast, analysis of another dataset from our laboratory (GEO: GSE135644) confirmed that YTHDF1 expression was reduced in AGS-EBV cells, whereas YTHDF3 showed no significant change (Figure 1D). We next validated these transcriptomic results using gastric cancer cell lines. EBV-positive cell lines (AGS-EBV, SNU719, YCCEL1) exhibited markedly lower YTHDF1 expression at both the mRNA and protein levels compared with EBV-negative cell lines (AGS, SNU216) (Figure 1E,F). Consistent with these results, patient-derived gastric cancer tissues (12 EBV-negative and 12 EBV-positive) showed significantly decreased YTHDF1 mRNA expression in EBV-associated gastric cancer (EBVaGC) compared with EBV-negative gastric cancer (EBVnGC) (Figure 1G). To explore the mechanism underlying YTHDF1 suppression in EBVaGC, we examined the potential role of EBV BART miRNAs. Target prediction analysis identified miR-BART15-5p and miR-BART19-3p as candidate regulators with seed sequence complementarity to the YTHDF1 3′ UTR (Supplementary Figure S3). Transfection of AGS cells with these miRNA mimics significantly reduced YTHDF1 mRNA expression compared with scramble controls, as shown by qRT-PCR (Figure 1H). Together, these results indicate that EBV infection suppresses YTHDF1 expression in gastric cancer, at least in part through regulation by EBV BART miRNAs.

### 3.2. Suppression of YTHDF1 Expression Inhibits Proliferation and Migration of Gastric Cancer Cells

**Figure 2:**
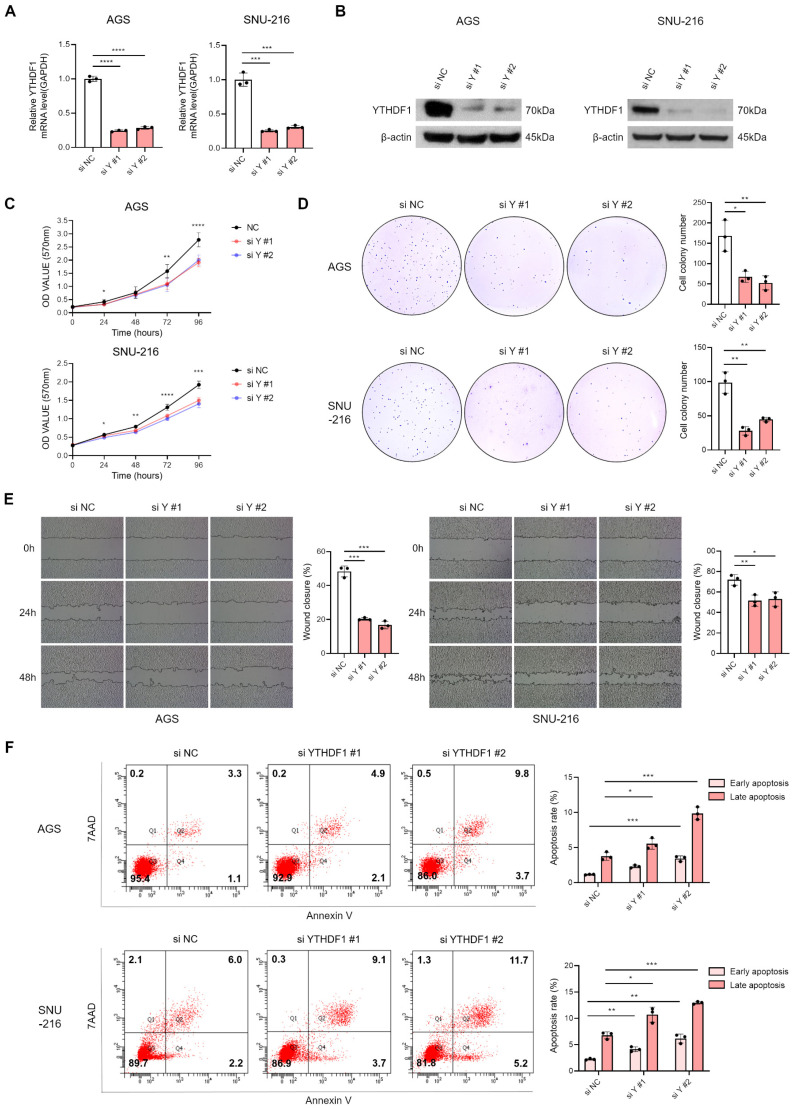
Effect of YTHDF1 knockdown on proliferation, migration, and apoptosis of EBV-negative gastric cancer cells. YTHDF1 expression was analyzed by qRT-PCR (**A**) and Western blot (**B**) following siRNA-mediated knockdown in EBV-negative gastric cancer cells. The effect of YTHDF1 knockdown on cell proliferation and colony-forming ability was assessed using MTT assay (**C**) and colony formation assay (**D**). Both experiments were independently performed three times, and representative results are shown in (**C**,**D**). (**E**) Wound healing assay was conducted to evaluate the migratory ability of EBV-negative gastric cancer cells following YTHDF1 knockdown. (**F**) Flow cytometry analysis was performed to assess apoptosis levels in EBV-negative gastric cancer cells following YTHDF1 knockdown. *p*-values: * *p* < 0.05; ** *p* < 0.01; *** *p* < 0.001; **** *p* < 0.0001.

To investigate the functional role of YTHDF1 in gastric cancer, we designed two independent siRNAs targeting different regions of YTHDF1 mRNA. qRT-PCR and Western blot analysis confirmed that both siRNAs effectively reduced YTHDF1 expression (Figure 2A,B). Functional assays revealed that YTHDF1 knockdown significantly inhibited cell proliferation, as shown by MTT and colony formation assays (Figure 2C,D). Wound healing assays further demonstrated that YTHDF1 depletion impaired the migratory capacity of EBV-negative gastric cancer cells (Figure 2E). In addition, YTHDF1 silencing markedly increased apoptosis, as assessed by flow cytometry (Figure 2F). These results collectively indicate that YTHDF1 is essential for maintaining the malignant properties of gastric cancer cells.

### 3.3. Overexpression of YTHDF1 Promotes Proliferation and Migration of Gastric Cancer Cells

**Figure 3:**
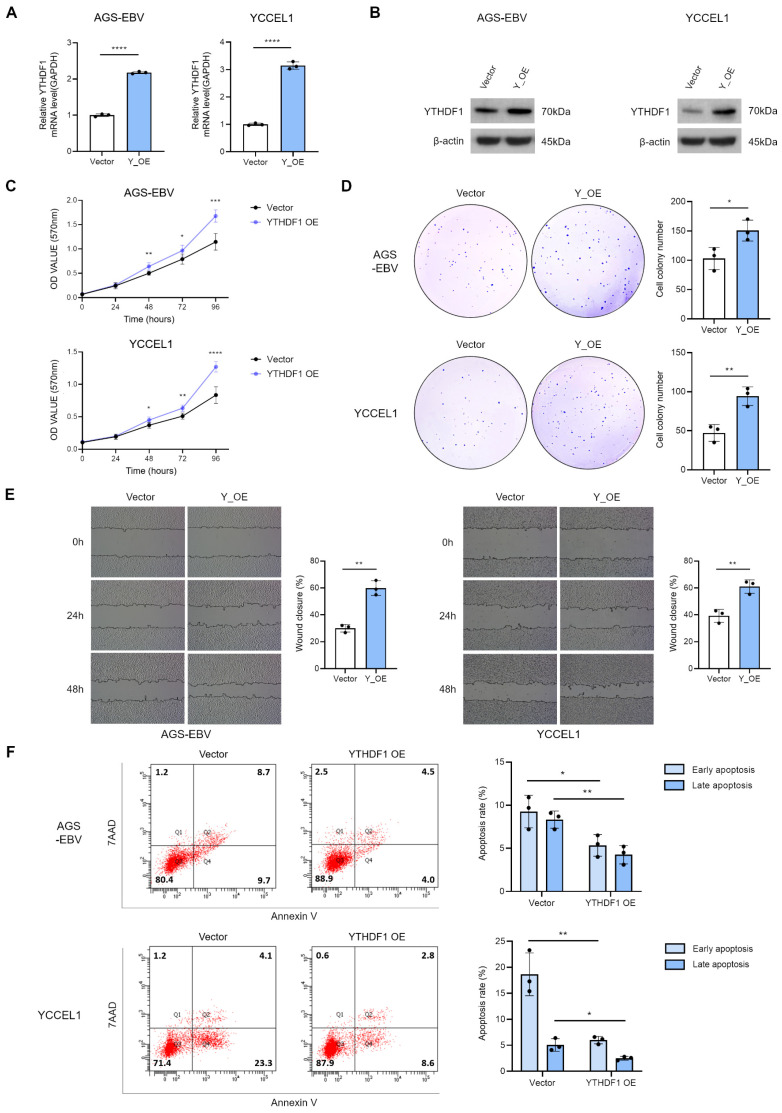
Effect of YTHDF1 overexpression on proliferation, migration, and apoptosis of EBV-positive gastric cancer cells. qRT-PCR (**A**) and Western blot (**B**) analyses confirmed increased YTHDF1 expression in EBV-positive gastric cancer cells transfected with a YTHDF1 overexpression vector. MTT assay (**C**) and colony formation assay (**D**) were performed to evaluate the effects of YTHDF1 overexpression on cell proliferation and oncogenicity. Both experiments were independently conducted three times, and representative results are shown in (**C**,**D**). (**E**) Wound healing assay was conducted to assess the impact of YTHDF1 overexpression on the migratory ability of EBV-positive gastric cancer cells. (**F**) Flow cytometry analysis was performed to evaluate the effect of YTHDF1 overexpression on apoptosis in EBV-positive gastric cancer cells. *p*-values: * *p* < 0.05; ** *p* < 0.01; *** *p* < 0.001; **** *p* < 0.0001.

To assess the effect of YTHDF1 on the tumorigenic properties, we generated a YTHDF1 overexpression vector and introduced it into two EBV-positive gastric cancer cell lines. Successful overexpression was confirmed by qRT-PCR and Western blot analyses (Figure 3A,B). MTT and colony formation assays demonstrated that YTHDF1 overexpression significantly enhanced cell proliferation (Figure 3C,D). Similarly, wound healing assays revealed that YTHDF1 overexpression markedly promoted cell migration (Figure 3E). Flow cytometric analysis following 7-AAD and Annexin V staining showed that YTHDF1 overexpression significantly reduced apoptosis in EBV-positive gastric cancer cells (Figure 3F). Together, these findings indicate that YTHDF1 acts as a critical regulator that promotes the malignant phenotype of gastric cancer by enhancing cell proliferation and migration.

### 3.4. EBV Infection Modulates the m6A Methylation Pattern of TSC22D1 in Gastric Cancer Cells

**Figure 4:**
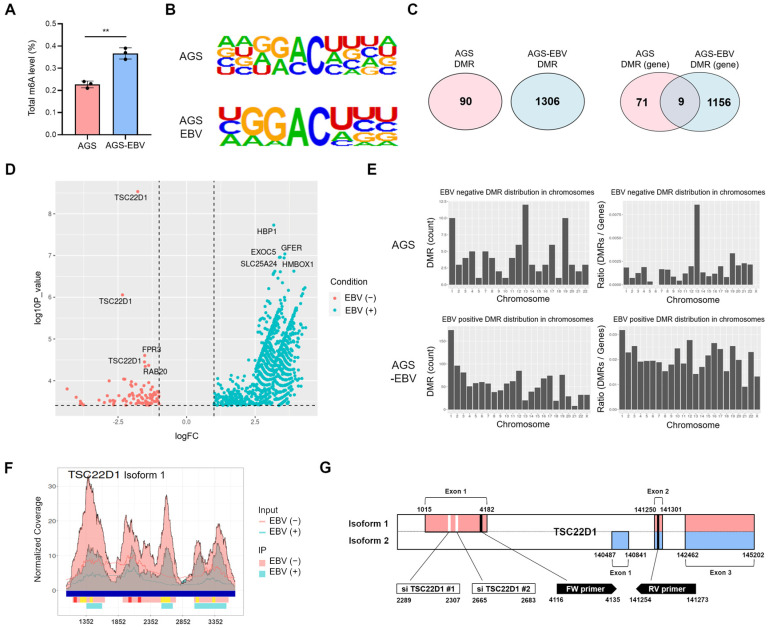
m6A methylation changes in gastric cancer cells upon EBV infection. (**A**) Quantification of m6A RNA methylation levels in AGS and AGS-EBV cells. (**B**) MeRIP sequencing identified m6A motif sequences (DRACH; D = G/A/U, R = G/A, H = A/U/C) in AGS and AGS-EBV cells. (**C**) Venn diagram showing differentially methylated regions (DMRs) and methylated genes in AGS and AGS-EBV cells. (**D**) Volcano plot visualizing differentially methylated genes in AGS and AGS-EBV cells. Red and blue dots represent EBV-negative and EBV-positive conditions, respectively. Vertical dashed lines indicate the log2 fold change thresholds (±1), and the horizontal dashed line indicates the significance cutoff (FDR < 0.05). (**E**) Chromosomal distribution of DMRs in AGS and AGS-EBV cells. (**F**) Composite visualization of DMRs and m6A methylation peaks across the TSC22D1 locus in AGS and AGS-EBV cells. Blue bars indicate exons on the reference genome. Pink and light-blue peaks represent m6A-enriched regions in AGS and AGS-EBV cells, respectively. Red bars denote AGS-specific DMRs, which are present only in AGS cells and absent in AGS-EBV cells, whereas yellow bars indicate shared DMRs detected in both cell types but exhibiting reduced peak intensity following EBV infection. The x-axis corresponds to nucleotide positions along the TSC22D1 gene according to the NCBI reference sequence. (**G**) Gene structures of TSC22D1 isoform 1 and isoform 2 are illustrated. In this study, the siRNAs and the primers for qRT-PCR used in this study were designed to target isoform 1 specific regions, as indicated in the figure. *p*-values: ** *p* < 0.01.

**Figure 5:**
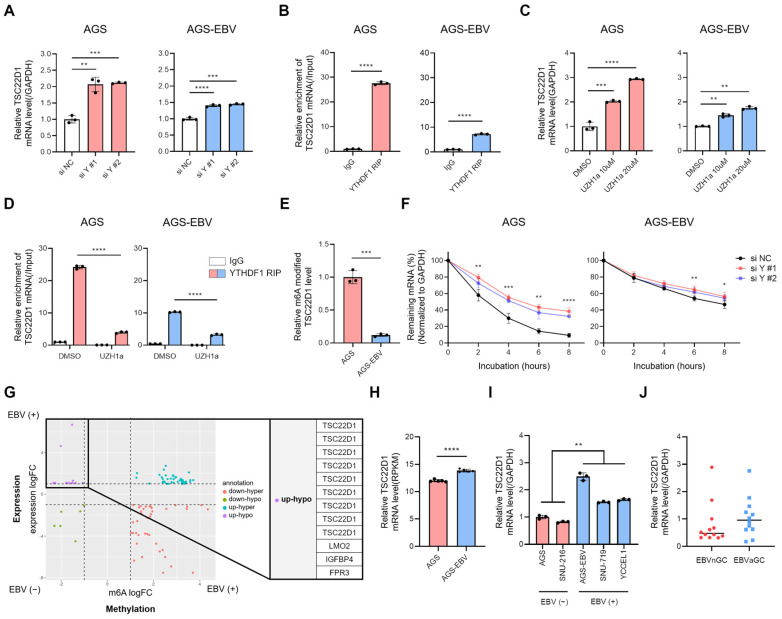
YTHDF1 regulates TSC22D1 mRNA stability in an m6A-dependent manner. (**A**) qRT-PCR analysis of TSC22D1 mRNA levels following YTHDF1 knockdown. (**B**) RIP-qPCR using a YTHDF1 antibody was performed to determine the direct interaction between YTHDF1 and TSC22D1 mRNA. Data were quantified using ΔCt values relative to input RNA. (**C**) qRT-PCR analysis of TSC22D1 mRNA levels after treatment with the METTL3 inhibitor UZH1a. (**D**) RIP-qPCR was conducted to assess the change in YTHDF1-TSC22D1 mRNA interaction following UZH1a treatment. The data were quantified as ΔCt values relative to input RNA. (**E**) m6A immunoprecipitation (IP) followed by qRT-PCR was performed to analyze the m6A methylation levels of TSC22D1 mRNA in AGS and AGS-EBV cells. (**F**) RNA stability assay using Actinomycin D was conducted to evaluate the effect of YTHDF1 knockdown on TSC22D1 mRNA stability. (**G**) Integration of differentially expressed genes (DEGs; RNA-seq) and differentially methylated regions (DMRs; MeRIP-seq) between AGS and AGS-EBV cells. The x-axis indicates changes in m6A methylation (m6A logFC), and the y-axis indicates changes in gene expression (expression logFC). Genes in the upregulated–hypomethylated (up-hypo) quadrant, showing increased expression with reduced m6A modification, are highlighted. Vertical dashed lines indicate the thresholds used to define changes in m6A methylation (m6A logFC), and the horizontal dashed line indicates the threshold for gene expression changes (expression logFC). These cutoff lines divide the plot into quadrants used to identify combined methylation–expression patterns. (**H**) TSC22D1 mRNA expression levels were compared between AGS and AGS-EBV cells using microarray data generated in our laboratory (GEO: GSE135644). (**I**) qRT-PCR analysis of TSC22D1 mRNA expression in EBV-negative (AGS, SNU216) and EBV-positive (SNU719, YCCEL1) gastric cancer cell lines. (**J**) Tissue-level validation of TSC22D1 expression using primary human gastric cancer specimens. qRT-PCR was performed to compare TSC22D1 mRNA levels between EBV-negative and EBV-positive tumors. *p*-values: * *p* < 0.05; ** *p* < 0.01; *** *p* < 0.001; **** *p* < 0.0001.

**Figure 6:**
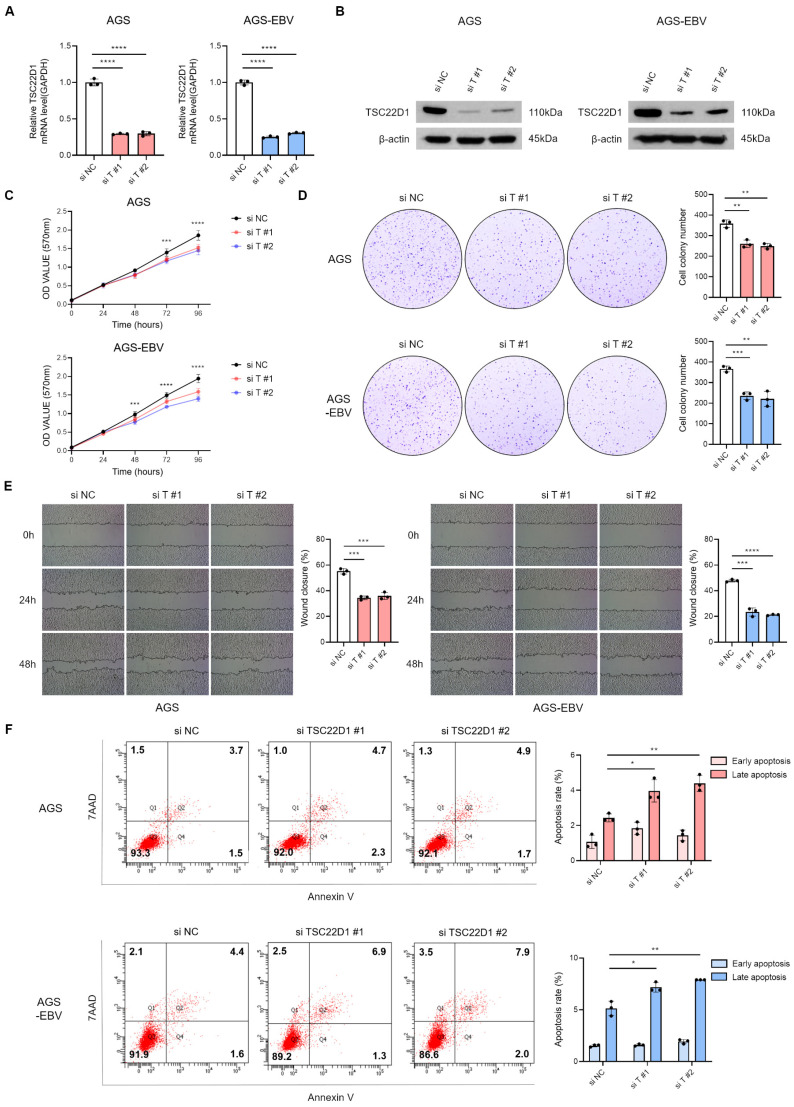
Functional impact of TSC22D1 knockdown in gastric cancer cells. qRT-PCR (**A**) and Western blot (**B**) analyses were performed to assess TSC22D1 expression levels following siRNA-mediated TSC22D1 knockdown in AGS and AGS-EBV cells. MTT assay (**C**) and colony formation assay (**D**) were conducted to assess the impact of TSC22D1 knockdown on cell proliferation and oncogenicity. Both experiments were independently performed three times, and representative results are shown in (**C**,**D**). (**E**) Wound healing assay was performed to assess the effect of TSC22D1 knockdown on cell migration in AGS and AGS-EBV cells. (**F**) Flow cytometry analysis using Annexin V/7-AAD staining was conducted to evaluate apoptosis following TSC22D1 knockdown in AGS and AGS-EBV cells. *p*-values: * *p* < 0.05; ** *p* < 0.01; *** *p* < 0.001; **** *p* < 0.0001.

**Figure 7:**
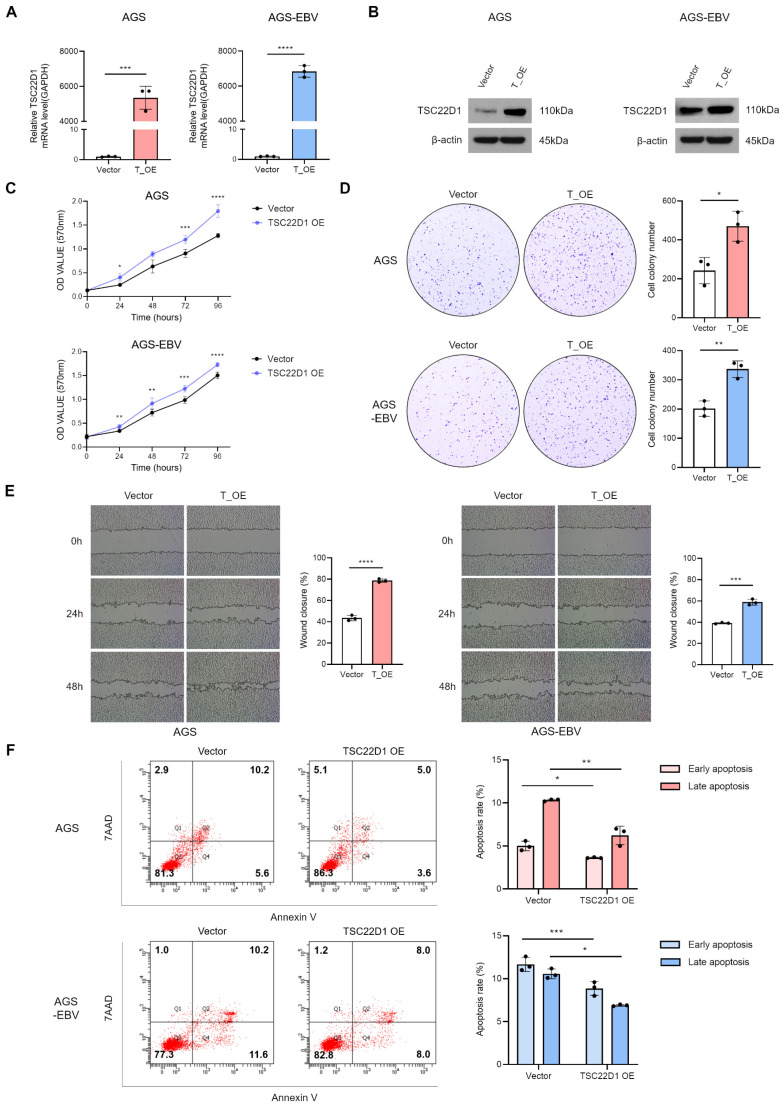
Functional impact of TSC22D1 overexpression in gastric cancer cells. TSC22D1 isoform 1 was overexpressed in AGS and AGS-EBV cells, and its expression levels were confirmed by qRT-PCR (**A**) and Western blot analysis (**B**). MTT assay (**C**) and colony formation assay (**D**) were performed to evaluate the effects of TSC22D1 overexpression on cell proliferation. (**E**) Wound healing assay was conducted to assess the impact of TSC22D1 overexpression on cell migration. (**F**) Flow cytometry analysis using Annexin V/7-AAD staining was carried out to measure the level of apoptosis. *p*-values: * *p* < 0.05; ** *p* < 0.01; *** *p* < 0.001; **** *p* < 0.0001.

Given the role of YTHDF1 as an mRNA m6A reader, we first compared global m6A methylation levels between AGS and AGS-EBV cells. AGS-EBV cells exhibited significantly higher overall m6A methylation than AGS cells (Figure 4A). To identify genes whose m6A methylation is influenced by EBV infection, we performed m6A RNA immunoprecipitation sequencing (MeRIP-seq) [28]. Motif analysis confirmed the enrichment of the consensus DRACH sequence (D = G/A/U, R = G/A, H = A/U/C) within m6A peak regions in both cell types (Figure 4B). Differentially methylated region (DMR) analysis revealed 90 DMRs in AGS cells and 1306 DMRs in AGS-EBV cells, corresponding to 71 and 1156 methylated genes, respectively (Figure 4C). These findings indicate that EBV infection induces substantial alterations in m6A modification, with both the number and location of modified regions differing depending on EBV status. Volcano plot visualization identified the top five genes with the most significant changes, including TSC22 domain family protein 1 (TSC22D1), FPR3, and RAB20 in AGS cells, and HBP1, EXOC5, GFER, SLC25A24, and HMBOX1 in AGS-EBV cells (Figure 4D). Chromosomal distribution analysis showed a notable enrichment of DMRs on chromosome 13 in AGS cells, eight of which were mapped to the TSC22D1 gene (Figure 4E). Integrated analysis of DMRs and m6A peaks revealed two distinct categories of m6A-modified regions within TSC22D1 (Figure 4F and Supplementary Figure S1). Three regions showed strong m6A peaks exclusively in AGS cells but were absent in AGS-EBV cells, whereas five additional regions were detected in both cell types but displayed markedly reduced peak intensity following EBV infection. These findings indicate that EBV infection selectively diminishes m6A methylation on TSC22D1. Among the multiple TSC22D1 isoforms, m6A peaks were specific to isoform 1 (Figure 4G). Therefore, all subsequent functional analyses-including siRNA knockdown and qRT-PCR- were performed using isoform 1-specific regions (Figure 5, Figure 6 and Figure 7). Overall, these findings demonstrate that EBV infection alters both global and locus-specific patterns of m6A methylation in gastric cancer cells. This alteration likely contributes to the regulation of gene expression and the modulation of tumor cell phenotypes.

### 3.5. YTHDF1 Binds to TSC22D1 mRNA and Regulates Its Stability in an m6A-Dependent Manner

Given that YTHDF1 is an m6A reader protein capable of influencing mRNA stability, we examined whether it regulates TSC22D1 expression. Knockdown of YTHDF1 using two independent siRNAs in both AGS and AGS-EBV cells led to an increase in TSC22D1 mRNA levels, with a greater fold change observed in AGS cells compared to AGS-EBV cells (Figure 5A). This difference likely reflects the higher basal expression of YTHDF1 in AGS cells and the stronger suppressive effect of its knockdown. Silencing of TSC22D1 did not alter YTHDF1 mRNA levels, indicating that TSC22D1 does not reciprocally regulate YTHDF1 (Supplementary Figure S2). To determine whether EBV miR-BART15-5p or miR-BART19-3p directly regulates TSC22D1 expression, we transfected AGS cells with each mimic and assessed TSC22D1 mRNA and protein levels. Neither mimic altered TSC22D1 expression (Supplementary Figure S4), indicating that these EBV miRNAs do not directly modulate TSC22D1. RNA immunoprecipitation (RIP) using a YTHDF1 antibody confirmed a direct interaction between YTHDF1 and TSC22D1 mRNA, with approximately 25-fold enrichment in AGS cells and 8-fold enrichment in AGS-EBV cells (Figure 5B). To access the role of m6A modification, cells were treated with the METTL3 inhibitor UZH1a. This treatment increased TSC22D1 mRNA levels in both cell types, although the fold change was smaller in AGS-EBV compared to AGS (Figure 5C). RIP assays following UZH1a treatment showed reduced binding of TSC22D1 mRNA to YTHDF1 (~ 6-fold in AGS; ~ 3-fold in AGS-EBV) (Figure 5D), consistent with m6A-dependent recognition. RIP using an m6A antibody further revealed that m6A-modified TSC22D1 mRNA was approximately 10-fold lower in AGS-EBV compared to AGS cells (Figure 5E). RNA stability assays using actinomycin D demonstrated that YTHDF1 knockdown increased TSC22D1 mRNA stability in both cell lines. In AGS cells, TSC22D1 mRNA was initially unstable but became markedly stabilized upon YTHDF1 silencing. In AGS-EBV cells, basal TSC22D1 mRNA stability was higher and showed only a modest increase after YTHDF1 knockdown (Figure 5F). Integrated analysis of DEGs and DMRs categorized TSC22D1 and several other genes as “up-hypo”, characterized by increased expression and decreased methylation upon EBV infection (Figure 5G and Supplementary Table S1). This suggests that EBV may enhance expression of these genes by relieving m6A-dependent repression. Consistently, microarray data (GEO: GSE135644) confirmed that TSC22D1 mRNA levels were significantly higher in AGS-EBV compared with AGS cells (Figure 5H). Further validation using qRT-PCR showed that TSC22D1 expression was significantly higher in EBV-positive cell lines (SNU719 and YCCEL1) compared with EBV-negative cell lines (AGS and SNU216) (Figure 5I). As an additional tissue-level validation, TSC22D1 mRNA expression was measured in primary gastric cancer tissues. Although substantial inter-patient variability was observed, EBV-positive tumors showed a consistent trend toward higher TSC22D1 expression compared with EBV-negative tumors (Figure 5J).

### 3.6. Silencing of TSC22D1 Inhibits Proliferation and Migration of Gastric Cancer Cells

To investigate the functional role of TSC22D1 in gastric cancer, isoform 1-specific siRNAs targeting the regions shown in Figure 4G were designed to knockdown TSC22D1. qRT-PCR (Figure 6A) and Western blot analysis (Figure 6B) confirmed efficient reduction in TSC22D1 expression with both siRNAs. MTT and colony formation assays revealed that TSC22D1 knockdown significantly suppressed proliferation in both AGS and AGS-EBV cells (Figure 6C,D). Wound healing assays further demonstrated that silencing TSC22D1 impaired cell migratory capacity (Figure 6E). In addition, flow cytometric analysis using Annexin V/7-AAD staining showed a significant increase in apoptosis following TSC22D1 knockdown (Figure 6F). These results indicate that TSC22D1 promotes gastric cancer cell survival, proliferation, and migration.

### 3.7. Overexpression of TSC22D1 Promotes Proliferation and Migration of Gastric Cancer Cells

To further elucidate the biological function of TSC22D1, isoform 1 was overexpressed in AGS and AGS-EBV cells. Successful overexpression was confirmed by qRT-PCR and Western blot analysis (Figure 7A,B). Functional assays revealed that TSC22D1 overexpression significantly enhanced cell proliferation and colony formation in both cell lines (Figure 7C,D). Wound healing assays demonstrated that overexpression accelerated cell migration (Figure 7E). Additionally, flow cytometric analysis using Annexin V/7-AAD staining showed a marked reduction in apoptosis following TSC22D1 overexpression (Figure 7F). These results indicate that TSC22D1 isoform 1 promotes proliferation and migration while inhibiting apoptosis in gastric cancer cells.

## 4. Discussion

In this study, we identified a novel regulatory mechanism by which Epstein–Barr virus (EBV) influences gastric cancer progression through modulation of N6-methyladenosine (m6A) epitranscriptomic pathways. RNA sequencing and microarray analyses demonstrated that EBV infection significantly suppresses the expression of the m6A reader protein YTHDF1 in gastric cancer cell lines and patient samples. Mechanistically, this suppression is at least partially mediated by EBV-encoded BART miRNAs, including miR-BART15-5p and miR-BART19-3p. Although the reduction in YTHDF1 mRNA by EBV BART miRNA mimics was modest, such small yet significant changes fall within the typical regulatory range of miRNA-mediated repression [29]. Because miRNAs often fine-tune gene expression through subtle modulation rather than large fold-changes, our findings support the idea that EBV employs miRNA-based mechanisms to exert precise post-transcriptional control in gastric cancer cells. Functionally, YTHDF1 knockdown inhibited cell proliferation, migration, and survival, while its overexpression enhanced these malignant properties. m6A-RIP sequencing revealed that EBV infection extensively remodels the host m6A landscape, including a marked reduction in m6A methylation of TSC22D1 isoform 1, a gene shown to promote gastric cancer cell growth and survival. Loss of m6A methylation on TSC22D1 led to decreased YTHDF1 binding and increased mRNA stability, indicating that EBV regulates gene expression through both transcriptional and epitranscriptomic mechanisms.

These findings offer key insights into how EBV co-opts the host epitranscriptome to facilitate oncogenesis. Notably, EBV infection not only increases global m6A levels but also induces transcript-specific hypomethylation at key oncogenic targets such as TSC22D1, suggesting that the virus can fine-tune gene regulation by modulating methylation patterns at select loci. The mechanism behind this selective m6A regulation likely involves RNA secondary structures, local sequence contexts including DRACH motifs, and the substrate specificity of the m6A methyltransferase complex [30]. Similar virus-driven reprogramming of m6A patterns has been reported in Kaposi’s sarcoma-associated herpesvirus (KSHV) and Flavivirus infections, where some transcripts are hypomethylated despite an overall increase in m6A [31,32,33]. Our study further supports this model, as TSC22D1 was found to be hypomethylated and overexpressed specifically in EBV-infected cells.

YTHDF1 is known to enhance translation and stability of oncogenic transcripts in several cancers, including liver, breast, ovarian, colorectal, and hematologic malignancies [34,35,36,37,38,39]. In gastric cancer, elevated YTHDF1 has been associated with tumor growth and immune evasion [40]. Interestingly, while YTHDF1 generally stabilizes or promotes translation of its targets, emerging evidence reveals it can also mediate transcript degradation via mechanisms involving AGO2 or miRNA complexes [41,42,43]. Our data reveal a similar duality: YTHDF1 binding to TSC22D1 leads to reduced mRNA stability, and this repression is relieved upon EBV-induced m6A hypomethylation and YTHDF1 downregulation, allowing TSC22D1 to accumulate and exert pro-tumorigenic effects. This finding emphasizes that the YTHDF1 function is transcript-specific and influenced by the epitranscriptomic context of each target. In addition, EBV-positive AGS-EBV cells exhibited a slightly slower basal proliferative rate compared with parental AGS cells, consistent with previous observations in EBV-associated gastric cancer models [44]. This growth pattern suggests that the EBV-induced stabilization of TSC22D1 may provide a supportive proliferative advantage that contributes to oncogenic progression in EBV-infected cells.

TSC22D1 is a multifunctional transcriptional regulator involved in key signaling pathways such as TGF-β, MAPK, and p53, with diverse roles depending on isoform and cellular context [45]. While isoform 2 has been described as a tumor suppressor [46,47], our data demonstrate that isoform 1 promotes proliferation and migration while inhibiting apoptosis in gastric cancer cells, consistent with its oncogenic function reported in other malignancies [48,49]. Importantly, this is the first study to show that TSC22D1 expression is regulated through m6A modification and that EBV infection specifically reduces m6A on TSC22D1 isoform 1, enhancing its stability.

**Figure 8:**
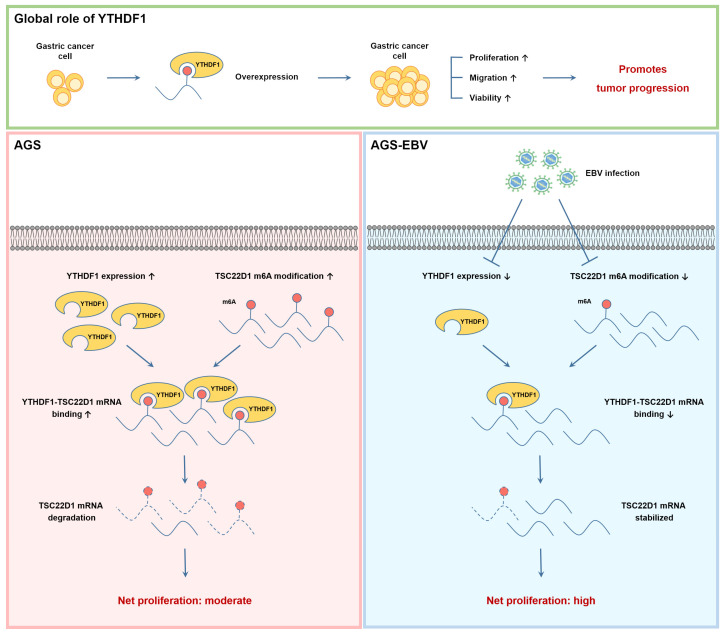
Schematic representation of the global and EBV-dependent regulation of TSC22D1 by YTHDF1 in gastric cancer cells. (**Top**) panel: YTHDF1 overexpression enhances proliferation, migration, and viability in gastric cancer cells, thereby promoting tumor progression. (**Bottom**) panels: In EBV-negative (AGS) cells (**left**), high YTHDF1 expression and elevated m6A methylation on TSC22D1 mRNA increase YTHDF1–TSC22D1 binding, leading to mRNA destabilization and reduced TSC22D1 expression, resulting in moderate net proliferation. In EBV-positive (AGS-EBV) cells (**right**), EBV infection decreases YTHDF1 expression and reduces m6A modification on TSC22D1 mRNA, resulting in diminished YTHDF1 binding and increased mRNA stability. Elevated TSC22D1 expression enhances tumorigenic potential, leading to net high proliferation.

The regulatory axis between YTHDF1 and TSC22D1 highlights a previously unrecognized mechanism through which EBV promotes gastric cancer progression. The relatively modest effects of YTHDF1 knockdown or METTL3 inhibition on TSC22D1 levels in EBV-positive cells further suggest that the m6A landscape in these cells is already reprogrammed, limiting the impact of additional m6A modulation. These observations underscore the importance of virus-induced baseline shifts in epitranscriptomic regulation and how they shape cellular responsiveness. Collectively, these findings support a mechanistic model illustrating how EBV modulates the YTHDF1–TSC22D1 axis in gastric cancer cells (Figure 8).

From a clinical standpoint, our findings offer several avenues for therapeutic exploration. First, the YTHDF1–TSC22D1 axis represents a novel target for disrupting EBV-driven oncogenesis. Therapeutic strategies aimed at restoring YTHDF1 function or reducing TSC22D1 expression may suppress tumor growth, particularly in EBV-associated gastric cancer (EBVaGC), which currently lacks targeted therapies. Second, YTHDF1 and TSC22D1 may serve as molecular biomarkers for stratifying EBV-positive versus EBV-negative gastric tumors or for predicting therapeutic response.

In conclusion, our study reveals a novel epitranscriptomic mechanism through which EBV promotes gastric cancer, involving miRNA-mediated suppression of YTHDF1 and selective hypomethylation of TSC22D1 mRNA. These findings highlight the complexity of m6A regulation in viral oncogenesis and identify potential molecular targets for therapeutic intervention in EBV-associated tumors.

## Figures and Tables

**Table 1:** Primer sequences used for qRT-PCR.

Gene	Forward (5′ → 3′)	Reverse (5′ → 3′)
GAPDH	ATGGGGAAGGTGAAGGTCG	GGGGTCATTGATGGCAACAATA
YTHDF1	CGACGACTTTGCTCACTACGA	CTGGTTCGCCCTCATTGTTT
TSC22D1	TTCCTAGTGCTGCTGGTGTG	TTCCTAGTGCTGCTGGTGTG

**Table 2:** siRNA sequences used for gene silencing.

siRNA	Sense (5′ → 3′)	Antisense (5′ → 3′)
Control	UUCUCCGAACGUGUCACGU	ACGUGACACGUUCGGAGAA
si YTHDF1 #1	CCGAAAGAGUUUGAGUGGA	UCCACUCAAACUCUUUCGG
si YTHDF1 #2	GCUCCAUUAAGUACUCCAU	AUGGAGUACUUAAUGGAGC
si TSC22D1 #1	GAGCAGGAACAACAGUGAUTT	AUCACUGUUGUUCCUGCUCTT
si TSC22D1 #2	ACAAGGAGUAGAACCAGUATT	UACUGGUUCUACUCCUUGUTT

## Data Availability

The data presented in this study are available from the corresponding author upon reasonable request. The data are not publicly available because they are stored locally by the authors and have not been deposited in a public repository.

## Supplementary Materials

The following supporting information can be downloaded at: https://www.mdpi.com/article/10.3390/microorganisms13122820/s1, Figure S1: Differential m6A methylation patterns in AGS and AGS-EBV cells; Figure S2: TSC22D1 knockdown does not affect YTHDF1 expression; Figure S3: Seed-match alignment of EBV miR-BART15-5p and miR-BART19-3p with the YTHDF1 3′UTR; Figure S4: miR-BART15-5p and miR-BART19-3p mimics do not alter TSC22D1 expression in AGS cells; Table S1: Conjoint analysis of DEGs and DMRs in AGS and AGS-EBV cells.
