# Supplementary material for: Epstein–Barr Virus Promotes Gastric Cancer Progression by Modulating m6A-Dependent YTHDF1–TSC22D1 Axis
Source: Microorganisms. 2025 Dec 11;13(12):2820. doi: 10.3390/microorganisms13122820 (PMC12736373; doi:10.3390/microorganisms13122820)
Supplement: Supplementary file 1 [file microorganisms-13-02820-s001.zip › microorganisms-3997439-supplementary.pdf]

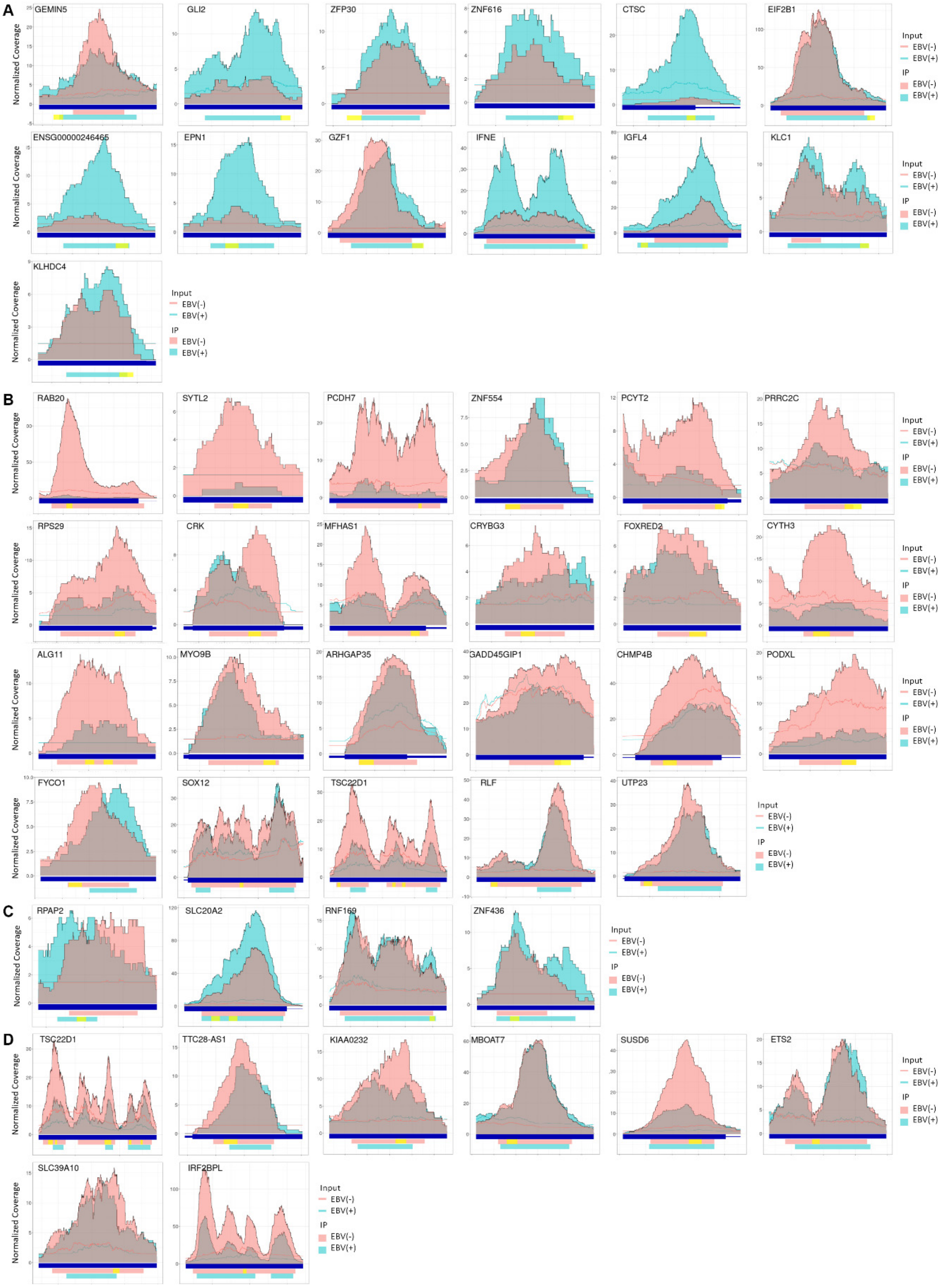
A
Normalized Coverage
GEMIN5
GLI2
ZFP30
ZNF616
CTSC
EIF2B1
ENSG00000246465
EPN1
GZF1
IFNE
IGFL4
KLC1
KLHDC4
Input
EBV(-)
EBV(+)
IP
EBV(-)
EBV(+)
B
RAB20
SYTL2
PCDH7
ZNF554
PCYT2
PRRC2C
RPS29
CRK
MFHAS1
CRYBG3
FOXRED2
CYTH3
ALG11
MYO9B
ARHGAP35
GADD45GIP1
CHMP4B
PODXL
FYCO1
SOX12
TSC22D1
RLF
UTP23
C
RPAP2
SLC20A2
RNF169
ZNF436
D
TSC22D1
TTC28-AS1
KIAA0232
MBOAT7
SUSD6
ETS2
SLC39A10
IRF2BPL

Supplementary Figure S1. Differential m6A methylation patterns in AGS and AGS-EBV cells. (A) Genes with DMRs present only in AGS-EBV, where the m6A peak intensity is higher in AGS-EBV than in AGS. (B) Genes with DMRs present only in AGS, where the m6A peak intensity is higher in AGS than in AGS-EBV. (C) Genes with DMRs present in both AGS and AGS-EBV, but with higher m6A peak intensity in AGS-EBV than in AGS. (D) Genes with DMRs present in both AGS and AGS-EBV, but with higher m6A peak intensity in AGS than in AGS-EBV. A table summarizes gene names, chromosome locations, start positions, log2FC values, and p-values for the identified DMRs, with corresponding visual representations. Blue bars indicate exons in the reference genome, while pink and light blue peaks represent m6A-enriched regions in AGS and AGS-EBV cells, respectively. Yellow bars denote differentially methylated regions (DMRs).

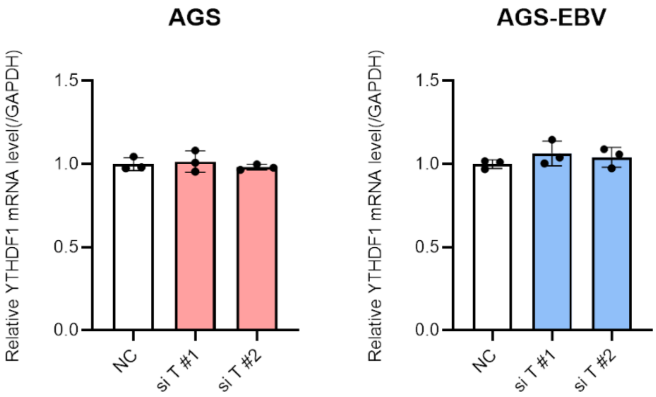

Supplementary Figure S2. TSC22D1 knockdown does not affect YTHDF1 expression. AGS and AGS-EBV cells were transfected with two different siRNAs targeting TSC22D1, and YTHDF1 mRNA expression levels wer e analyzed by qRT-PCR.

miR-BART 15-5p

```
target 5'              C       A 3'
                        CCACUGA
                        GGUGACU
miRNA  3' AGUUCCUUUGUUUU       G 5'
```

miR-BART 19-3p (site 1)

```
target 5' A         UAA         C 3'
           AGUA UCCU    AAACAAAG
           UCGU AGGG    UUUGUUUU
miRNA  3'     A    UUCG          5'
```

miR-BART 19-3p (site 2)

```
target 5'   U     GUUGAAU    AA        C 3'
             UUCCU       GGGU  AAACAAAA
             AAGGG       UUCG  UUUGUUUU
miRNA  3' UCGU                           5'
```

Supplementary Figure S3. Seed-match alignment of EBV miR-BART15-5p and miR-BART19-3p with the YTHDF1 3′UTR. miRNA sequences were obtained from miRBase (v22.1, accessed in February 2024), and putative target sites were predicted using RNAhybrid (v2.1.2, prediction performed in February 2024).

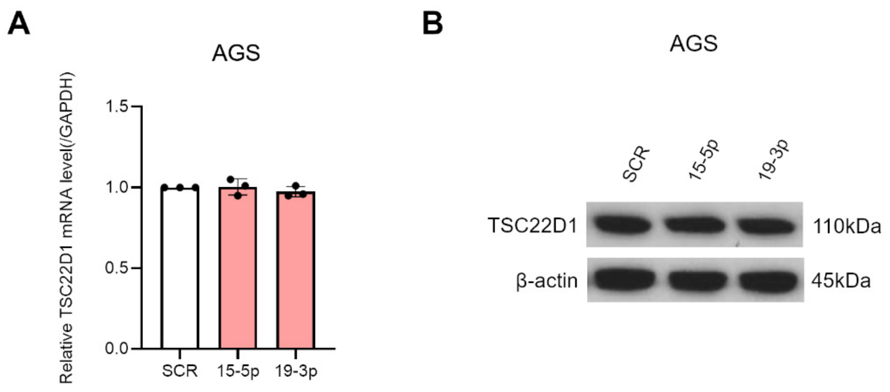

Supplementary Figure S4. miR-BART15-5p and miR-BART19-3p mimics do not alter TSC22D1 expression in AGS cells. (A) qRT-PCR analysis of TSC22D1 mRNA levels in AGS cells transfected with SCR control, miR-BART15-5p mimic, or miR-BART19-3p mimic. Data represent three independent biological replicates (n = 3). (B) Western blot analysis of TSC22D1 protein levels following mimic transfection. Representative blots from three independent experiments are shown. β-actin was used as a loading control.

Supplementary Table S1. Conjoint analysis of differentially expressed genes (DEGs) and differentially methylated regions (DMRs) in AGS and AGS-EBV cells.

	ENSG	Gene symbol	chromosome	start	end	DMR logFC	DEG logFC
• **up-hyper**	ENSG00000014914	MTMR11	1	149931483	149931532	3.38	1.13
	ENSG00000029153	ARNTL2	12	27423107	27423156	3.40	1.06
	ENSG00000032444	PNPLA6	19	7554992	7555041	2.48	1.15
	ENSG00000032444	PNPLA6	19	7554209	7554240	2.77	1.15
	ENSG00000032444	PNPLA6	19	7541003	7541051	2.40	1.15
	ENSG00000032444	PNPLA6	19	7540270	7540308	3.33	1.15
	ENSG00000049759	NEDD4L	18	58333821	58333870	1.81	1.03
	ENSG00000074047	GLI2	2	120991734	120991783	2.83	1.81
	ENSG00000074047	GLI2	2	120989634	120989683	2.53	1.81
	ENSG00000078018	MAP2	2	209730506	209730555	3.58	1.35
	ENSG00000081026	MAGI3	1	113651031	113651080	3.33	1.05
	ENSG00000088899	LZTS3	20	3167957	3168006	3.14	1.34
	ENSG00000095752	IL11	19	55365910	55365959	2.25	1.87
	ENSG00000099250	NRP1	10	33179514	33179563	1.28	2.03
	ENSG00000099326	MZF1	19	58568938	58568987	2.86	1.45
	ENSG00000100027	YPEL1	22	21698517	21698566	3.26	1.19
	ENSG00000105429	MEGF8	19	42350264	42350313	2.86	1.11
	ENSG00000108932	SLC16A6	17	68270839	68270878	2.35	1.64
	ENSG00000109861	CTSC	11	88326408	88326443	1.26	1.58
	ENSG00000116106	EPHA4	2	221418881	221418930	3.18	1.48
	ENSG00000116991	SIPA1L2	1	232515161	232515210	3.26	1.29
	ENSG00000121895	TMEM156	4	38993789	38993838	3.71	1.36
	ENSG00000134262	AP4B1	1	113902834	113902862	2.40	1.47
	ENSG00000140807	NKD1	16	50636991	50637040	3.22	1.00
	ENSG00000141424	SLC39A6	18	36108614	36108663	2.86	1.75
	ENSG00000141655	TNFRSF11A	18	62385194	62385243	3.58	1.19
	ENSG00000142408	CACNG8	19	53989116	53989165	2.44	1.22
	ENSG00000145246	ATP10D	4	47558040	47558089	2.74	1.50
	ENSG00000153898	MCOLN2	1	84926125	84926174	3.33	1.01
	ENSG00000161642	ZNF385A	12	54369672	54369721	2.97	1.64
	ENSG00000164684	ZNF704	8	80821449	80821498	3.22	1.07
	ENSG00000165802	NSMF	9	137448135	137448184	3.53	2.10
	ENSG00000167535	CACNB3	12	48828289	48828338	3.37	1.10
	ENSG00000180891	CUEDC1	17	57863259	57863308	2.56	1.01
	ENSG00000180891	CUEDC1	17	57872793	57872842	2.64	1.01
	ENSG00000183963	SMTN	22	31097013	31097062	2.40	1.19
	ENSG00000184995	IFNE	9	21481964	21482013	2.44	2.37
	ENSG00000186567	CEACAM19	19	44683457	44683506	3.26	1.35
	ENSG00000186567	CEACAM19	19	44684007	44684056	3.20	1.35
	ENSG00000197050	ZNF420	19	37127642	37127691	3.22	1.27
	ENSG00000204869	IGFL4	19	46057969	46058018	2.53	2.73
	ENSG00000225138	SLC9A3-AS1	5	478704	478753	3.40	1.21
	ENSG00000229807	XIST	X	73839174	73839223	3.04	1.20
	ENSG00000242221	PSG2	19	43071804	43071853	2.60	1.45
	ENSG00000246465	-	16	28286066	28286115	2.94	1.60

	ENSG	Gene symbol	chromosome	start	end	DMR logFC	DEG logFC
● **down-hyper**	ENSG00000008517	IL32	16	3067597	3067646	2.26	-5.48
	ENSG00000054983	GALC	14	87945601	87945650	1.02	-3.70
	ENSG00000054983	GALC	14	87959553	87959591	1.24	-3.70
	ENSG00000054983	GALC	14	87941457	87941506	1.10	-3.70
	ENSG00000068078	FGFR3	4	1804741	1804790	4.34	-1.44
	ENSG00000074416	MGLL	3	127690661	127690710	1.88	-1.20
	ENSG00000086548	CEACAM6	19	41772056	41772105	1.73	-3.78
	ENSG00000100077	GRK3	22	25723268	25723317	1.47	-1.86
	ENSG00000100934	SEC23A	14	39032879	39032928	1.92	-1.33
	ENSG00000101224	CDC25B	20	3801762	3801802	1.11	-1.04
	ENSG00000102854	MSLN	16	764907	764945	1.34	-3.90
	ENSG00000107719	PALD1	10	70566591	70566640	2.11	-4.72
	ENSG00000107719	PALD1	10	70566791	70566840	1.27	-4.72
	ENSG00000109072	VTN	17	28369520	28369569	2.76	-6.92
	ENSG00000111331	OAS3	12	112970766	112970815	1.84	-2.15
	ENSG00000113758	DBN1	5	177458477	177458526	1.81	-3.90
	ENSG00000114541	FRMD4B	3	69171783	69171832	1.11	-2.70
	ENSG00000125844	RRBP1	20	17621715	17621764	1.66	-1.19
	ENSG00000128567	PODXL	7	131503305	131503354	1.95	-1.65
	ENSG00000130600	H19	11	1996123	1996172	1.15	-6.35
	ENSG00000132623	ANKEF1	20	10038303	10038352	1.29	-2.58
	ENSG00000138835	RGS3	9	113507537	113507586	4.06	-1.05
	ENSG00000139629	GALNT6	12	51379684	51379733	1.88	-1.92
	ENSG00000140297	GCNT3	15	59619848	59619897	2.39	-5.33
	ENSG00000147065	MSN	X	65740845	65740894	1.63	-3.76
	ENSG00000151012	SLC7A11	4	138164542	138164591	1.56	-1.42
	ENSG00000151012	SLC7A11	4	138170792	138170841	3.43	-1.42
	ENSG00000151914	DST	6	56541858	56541907	2.94	-1.06
	ENSG00000165949	IFI27	14	94111688	94111737	1.45	-1.67
	ENSG00000176092	CRYBG2	1	26328804	26328853	1.74	-2.28
	ENSG00000196188	CTSE	1	206009897	206009946	1.50	-6.01
	ENSG00000196188	CTSE	1	206009047	206009096	2.50	-6.01
	ENSG00000197045	GMFB	14	54477380	54477429	1.83	-1.14
	ENSG00000197045	GMFB	14	54476530	54476579	1.43	-1.14
	ENSG00000198959	TGM2	20	38129933	38129982	3.70	-7.52
	ENSG00000198959	TGM2	20	38138305	38138354	2.41	-7.52
	ENSG00000203485	INF2	14	104703100	104703149	1.37	-1.26
	ENSG00000204370	SDHD	11	112095194	112095243	2.55	-1.40
	ENSG00000262246	CORO7	16	4408181	4408228	3.45	-1.20

	ENSG	Gene symbol	chromosome	start	end	DMR logFC	DEG logFC
• **up-hypo**	ENSG00000102804	TSC22D1	13	44574307	44574356	-2.25	1.09
	ENSG00000102804	TSC22D1	13	44573407	44573506	-2.34	1.09
	ENSG00000102804	TSC22D1	13	44573557	44573606	-1.54	1.09
	ENSG00000102804	TSC22D1	13	44574657	44574806	-1.78	1.09
	ENSG00000102804	TSC22D1	13	44573307	44573356	-1.06	1.09
	ENSG00000102804	TSC22D1	13	44575207	44575256	-1.38	1.09
	ENSG00000102804	TSC22D1	13	44576107	44576156	-1.76	1.09
	ENSG00000102804	TSC22D1	13	44574157	44574206	-1.66	1.09
	ENSG00000102804	TSC22D1	13	44575457	44575506	-1.51	1.09
	ENSG00000135363	LMO2	11	33859022	33859071	-2.01	4.63
	ENSG00000141753	IGFBP4	17	40443700	40443749	-1.49	1.30
	ENSG00000187474	FPR3	19	51825212	51825261	-1.53	6.68
• **down-hypo**	ENSG00000008256	CYTH3	7	6163134	6163183	-1.01	-1.09
	ENSG00000128567	PODXL	7	131500605	131500654	-1.18	-1.65
	ENSG00000137501	SYTL2	11	85724308	85724357	-2.29	-3.04
	ENSG00000139832	RAB20	13	110523623	110523672	-1.38	-4.50
	ENSG00000169851	PCDH7	4	30724065	30724114	-2.03	-3.05
	ENSG00000185813	PCYT2	17	81904880	81904929	-1.99	-1.69

DEGs and DMRs were concurrently analyzed to identify genes whose expression changes were associated with alterations in m6A methylation levels. Genes were categorized into four groups: (1) Up-Hyper: upreg ulated in AGS-EBV cells and exhibiting higher m6A methylation levels in AGS-EBV; (2) Up-Hypo: upregul ated in AGS-EBV but showing higher m6A levels in AGS; (3) Down-Hyper: downregulated in AGS-EBV w ith higher m6A methylation in AGS-EBV; and (4) Down-Hypo: downregulated in AGS-EBV and exhibiting higher m6A levels in AGS. DEGs and DMRs were identified using thresholds of $|\log_2$ fold change ($\log_2$FC)$| \geq 1$ and false discovery rate (FDR) < 0.05.
